# Recent Advances in Drug Discovery from South African Marine Invertebrates

**DOI:** 10.3390/md13106366

**Published:** 2015-10-14

**Authors:** Michael T. Davies-Coleman, Clinton G. L. Veale

**Affiliations:** 1 Department of Chemistry, University of the Western Cape, Robert Sobukwe Road, Bellville 7535, South Africa; 2 Faculty of Pharmacy, Rhodes University, Grahamstown 6140, South Africa; E-Mail: C.Veale@ru.ac.za

**Keywords:** cephalostatin, mandelalide, methicillin resistant *Staphylococcus aureus*, MRSA PK, bisindole alkaloids

## Abstract

Recent developments in marine drug discovery from three South African marine invertebrates, the tube worm *Cephalodiscus gilchristi*, the ascidian *Lissoclinum* sp. and the sponge *Topsentia pachastrelloides*, are presented. Recent reports of the bioactivity and synthesis of the anti-cancer secondary metabolites cephalostatin and mandelalides (from *C. gilchristi* and *Lissoclinum* sp., respectively) and various analogues are presented. The threat of drug-resistant pathogens, e.g., methicillin-resistant *Staphylococcus aureus* (MRSA), is assuming greater global significance, and medicinal chemistry strategies to exploit the potent MRSA PK inhibition, first revealed by two marine secondary metabolites, *cis*-3,4-dihydrohamacanthin B and bromodeoxytopsentin from *T. pachastrelloides*, are compared.

## 1. Introduction

**Figure 1:**
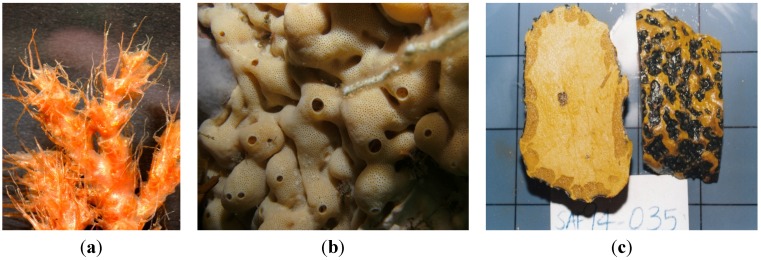
(**a**) *Cephalodiscus gilchristi* (photo: L. Lange); (**b**) *Lissoclinum* sp. (photo: S. Parker-Nance); (**c**) *Topsentia pachastrelloides* (photo: M. Davies-Coleman).

The plethora of intertidal and subtidal marine organisms inhabiting the *ca.* 26,000-km coastline of Africa provide a relatively untapped opportunity for the discovery of new bioactive secondary metabolites. Despite a concerted marine bio-discovery effort over the past four decades that has focused predominantly on South African marine invertebrates [1,2,3], only three South African marine invertebrates, *viz.* the tube worm *Cephalodiscus gilchristi*, the ascidian *Lissoclinum* sp. and the sponge *Topsentia pachastrelloides* (Figure 1), have afforded secondary metabolites whose biomedicinal potential is currently attracting international attention. Recent reports appearing in the chemical literature (June 2012–June 2015) pertaining to this cohort of secondary metabolites is overviewed. Not surprisingly, given the global problem of obtaining sufficient supplies of bioactive marine natural products from the ocean for further drug development [4], the bioactive secondary metabolites from these three South African marine invertebrates have been the subject of concerted synthetic programs geared towards producing sufficient amounts of either the natural product or potentially more bioactive analogues, for detailed biological and *in vitro* studies.

## 2. Tumor Growth Inhibiting Cephalostatins from the South African Marine Tube Worm *Cephalodiscus gilchristi*

**Figure 2:**
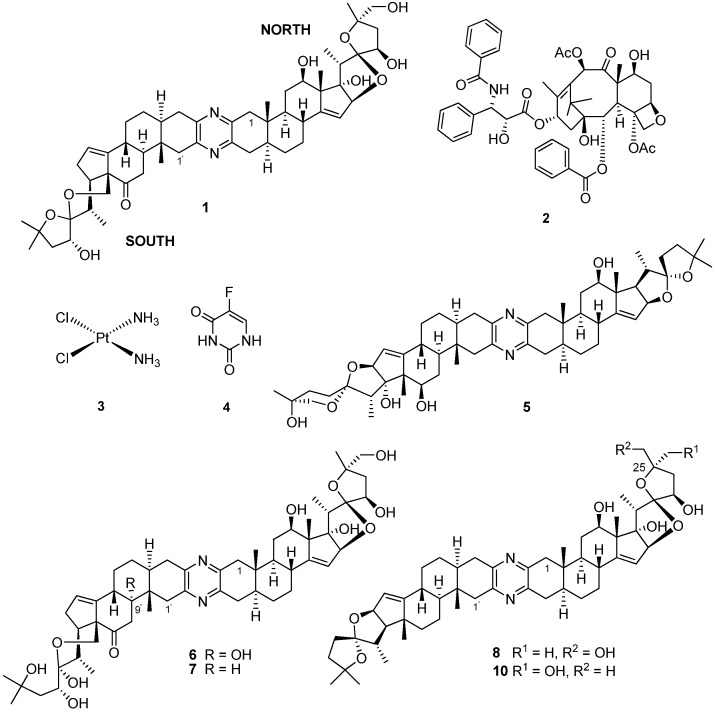
Chemical structures of compounds **1**–**8** and **10**.

The first large-scale collections of African marine invertebrates solely for the purpose of new drug discovery were coordinated by Professor G. R. Pettit of Arizona State University, USA, over three decades ago off South Africa’s temperate southern coast [5]. Cephalostatin 1 (**1**, Figure 2) was isolated in low yield (*ca.* 2.3 × 10^−7^%) from two separate and substantial SCUBA collections (166 and 450 kg (wet weight) collected in 1981 and 1990, respectively) of the hemichordate marine tube worm *Cephalodiscus gilchristi* (Figure 1a) [6]. Cephalostatin 1 has emerged as one of the most potent cell growth-inhibiting secondary metabolites ever screened by the U.S. National Cancer Institute (NCI) (ED_50_ 0.1–0.0001 pM in a P338 leukemia cell line) [6,7].

Of immediate interest to those exploring this compound’s tumor growth inhibitory activities was, first, the comparative GI_50_ values (quantification of the concentration required to inhibit cellular growth by 50%) of **1** (GI_50_ 1.2 nM) with commercially available anticancer drugs, e.g., taxol (**2**, GI_50_ 29 nM), cisplatin (**3**, GI_50_ 2000 nM) and 5-fluorouracil (**4**, 24,000 nM), and, second, the 275-times higher concentration of **1** required to kill 50% of cancer cells (LC_50_ 330 nM) relative to the amount required for 50% cell growth inhibition [6]. In addition, the application of the NCI’s COMPARE algorithm [8] to the GI_50_ data acquired for **1** indicated that this novel bis-steroidal pyrazine alkaloid possesses a unique mechanism of action against the proliferation of cancer cells in the NCI’s *in vitro* 60 cancer cell line screen, and therefore, not surprisingly, **1** is increasingly proving to be a valuable tool for the discovery of new apoptosis signaling pathways [9]. Vollmar and co-workers’ early studies into cephalostatin’s apoptotic mechanism of action established that **1** promotes the release of Smac (second mitochondria-derived activator of caspase) through the dissipation of mitochondrial membrane potential [6,9,10] as part of a novel apoptosome-independent, caspase-9-mediated apoptotic pathway [6]. Furthermore, Shair and co-workers have shown that **1** also selectively binds to oxysterol binding protein (OSBP) and OSBP-related protein 4L (ORP4L) [11] and drew attention to these proteins, whose role in cancer cell survival was little known at the time. A further eighteen naturally-occurring and semi-synthetic analogues of **1** have subsequently been reported (1988–2012) in the chemical and patent literature (e.g., U.S. Patents 4873245, 5047532, 5583224 and WO 8908655). The isolation, structure elucidation, synthesis and bioactivity of this cohort of cephalostatins has been comprehensively reviewed along with the closely-related bis-steroidal pyrazine alkaloids, the ritterazines, e.g., ritterazine G, (**5**) from the Japanese ascidian (tunicate), *Ritterella tokioka* [6]. Since the publication of Iglesias-Arteaga and Morzycki’s extensive review [6], the chemical structure of the twentieth member of the cephalostatin series, cephalostatin 20 (**6**), has recently been reported by Pettit *et al.* [12]. Compound **6**, the 9′-α-hydroxy analog of cephalostatin 9 (**7**), was isolated in low yield (1 × 10^7^%) from the combined bioactive (cytotoxic to P338 murine lymphocyte cells) fractions from the original extract of *C. gilchristi* [5] nearly a quarter of a century ago. Interestingly, the cell growth inhibitory activities of **6** and **7** against six human tumor cell lines was 100–1000-times less active than **1** in the same tumor cell panel, thus underlining the importance of an intact spirostanol structure in the southern unit of cephalostatins to the growth inhibition activities of these compounds [12].

**Figure 3:**
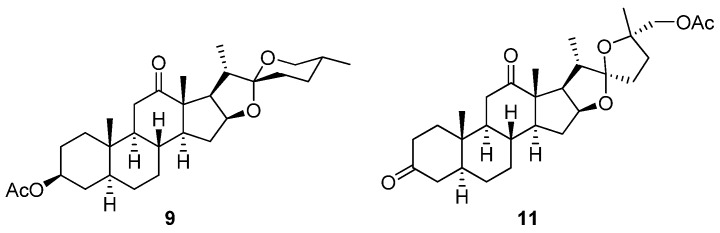
Chemical structures of synthetic intermediates **9** and **11**.

Significant effort [6,13,14] has been directed towards the total enantioselective syntheses of **1** over the last two decades. Following on from their first 65-step convergent total synthesis of **1** and potently active cephalostatin/ritterazine hybrids [15], Fuchs and co-workers have recently reported the first convergent total synthesis of 25-*epi* ritterostatin G_N_1_N_ **8** [16] from commercially available dihydroxyhecogenin acetate (**9**, Figure 3). Fuchs and co-workers identified the key step in their synthesis as a chiral ligand ((DHQ)_2_PHAL)-mediated dihydroxylation reaction, which introduced the 25-*epi* functionality into the north segment (analogous to the north unit of cephalostatin) [16]. Compound **8**, structurally incorporating the north units of both **1** and **5**, exhibited a mean GI_50_ (0.48 nM) in a panel of eight cancer cell lines and was 30-fold more active than ritterostatin (**10**), also screened in the same cell line panel [16].

The daunting synthetic challenges of cephalostatin molecular architecture continue to inspire the synthesis of simpler analogs with similar bioactivities to **1** [6]. The latest target in this series, [5.5]-spiroketal (**11**, Figure 3), which shares the steroidal scaffold of the northern hemisphere of **1** with an intact 1,6-dioxaspiro[5.5]nonane side chain, but with a diminished oxygenation pattern, was synthesized by Pettit *et al.* in seven steps from **9** in an overall 4.6% yield [17]. Although **11** and several synthetic precursors of this compound were not cytotoxic to P388 leukemia cells, Pettit *et al.* suggested that **9** was potentially useful as a synthetically-accessible starting point for further synthetic modification into both symmetrical and asymmetrical trisdecacyclic bis-steroidal pyrazine congeners of **1** [17] through well-established pyrazine ring construction protocols [16].

## 3. Synthesis and Revision of the Absolute Configuration of the Cytotoxic Mandelalides from the South African Marine Ascidian, *Lissoclinum* sp.

**Figure 4:**
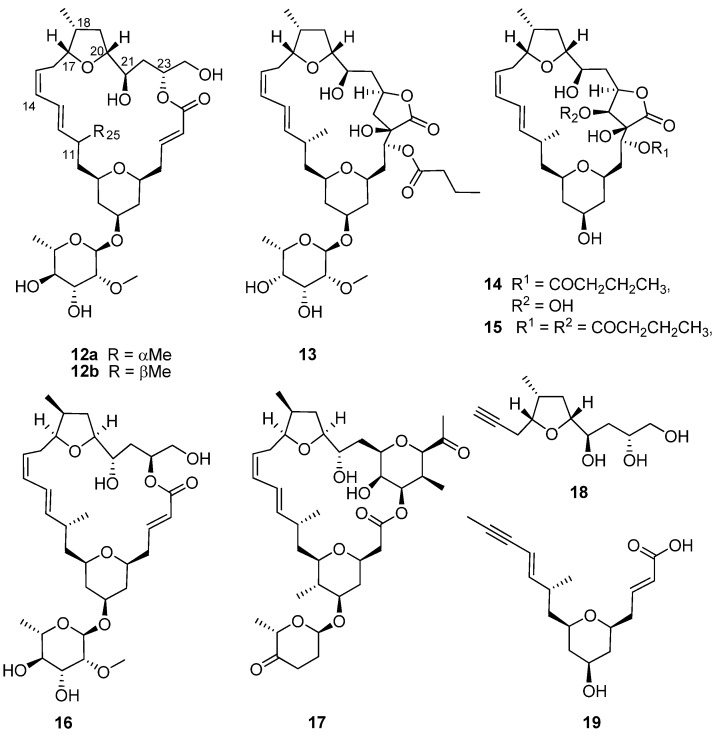
Chemical structures of compounds **12a**–**19**.

The encrusting colonial didemnid ascidian *Lissoclinum* sp. (Figure 1b) collected by SCUBA from Algoa Bay, on the southeast coast of South Africa, afforded sub-milligram (0.5–0.8 mg) quantities of the glycosylated, polyketide macrolides, the mandelalides A–D (**12a**, **13**–**15**, Figure 4). Mandelalides A and B exhibited potent low nanomolar cytotoxicity (IC_50_ 12 and 44 nM, respectively) against NCI-H460 lung cancer cells [18]. The relative configuration of the macrolide rings in **12a**, **13**–**15** was established through integration of ROESY data with homonuclear (^3^*J*_HH_) and heteronuclear (^2,3^*J*_CH_) coupling constants, while the absolute configurations of **12a** and **13** were extrapolated from the hydrolysis and subsequent chiral GC-MS analysis of the respective monosaccharide residues (2-*O*-methyl-6-dehydro-α-L-rhamnose and 2-*O*-methyl-6-dehydro-α-L-talose). The paucity of **12a**, **13**–**15** isolated from the MeOH-CH_2_Cl_2_ extract of *Lissoclinum* sp. (*i.e.*, 0.8 mg of **12a**) and difficulties encountered in the further supply of these compounds from their natural source implied that the synthesis of **12a** (the most active compound in the mandelalide series) would provide sufficient quantities of **12a** to explore the mechanism of *in vitro* cytotoxicity exhibited by this compound. As described below, the synthesis of **12a** and the diastereomer **16** by Willwacher *et al.* and Xu, Ye and co-workers revealed errors in the original assignment of the absolute configuration at positions C17, C18 C20, C21 and C23 and resulted in the correction of the chemical structure of mandelalide A (**12a**) to **16** [19,20].

In 2014, two years after the isolation of the mandelalides was first reported [18], Willwacher and Fürstner reported the first total synthesis of **12a** in a 4.5% overall yield [21]. They also noted the structural similarities between **12a** and madeirolide A (**17**, Figure 4), an equally scarce metabolite previously isolated from a marine sponge, *Leiodermatium* sp. [22,23], and ascribed the absence of anti-proliferative activity against pancreatic cancer cells reported for **17** to the structural differences between these two compounds. Anticipating in their proposed synthesis of **12a** that final closure of the macrolide ring, concomitant with insertion of the ∆^14^ *Z*-olefin, could be achieved with ring closing alkyne metathesis (RCAM), Willwacher and Fürstner successfully synthesized the two main building blocks (**18**) and (**19**) emerging from their retrosynthetic analysis of **12a**. Cobalt-catalyzed carbonylative epoxide opening and iridium-catalyzed two-directional Krische allylation were identified as key synthetic steps required for the synthesis of **18** and **19**, respectively, while the RCAM protocol would not have been possible without the use of a highly-selective molybdenum alkylidene complex catalyst; the first time that this catalyst has been successfully incorporated into a natural product total synthesis [21]. Finally, regioselective trimethylsilyl trifluoromethanesulfonate (TESOTf)-catalyzed rhamnosylation of the mandelalide aglycone proceeded smoothly to afford mandelalide A with the chemical structure **12a**, originally proposed by McPhail and co-workers [18].

Comparing the spectroscopic data acquired for their synthetic product with those of naturally-occurring mandelalide A (**16**), Willwacher and Fürstner noted significant chemical shift and coupling constant differences between the NMR datasets of synthetic **12a** and the natural product (Table 1 and Table 2). Initially, given the relative magnitude of the observed differences, their attention was focused on differences in the ^13^C chemical shift assigned to the C11 methine and the C25 methyl carbon atoms (Table 1) and discrepancies in the ^3^*J*_11,12_ coupling constants (Table 2; *J*_11,12_ = 9.7 and 7.6 Hz, respectively, for naturally-occurring mandelalide A and **12a**, respectively) associated with the C11 stereogenic center. However, synthesis of the 11-*epi*-diasteromer of **12a** (**12b**) did not provide clarity on the source of spectroscopic differences between the synthetic and natural products, and Willwacher and Fürstner were at a loss to explain where the anomalies resided in the proposed structure of **12a** [21]. Reddy *et al.* [24] also tackled the synthesis of the aglycone of **12a**, reflected in the original structure proposed by McPhail and co-workers [18]. Their 32-step synthesis afforded the putative mandelalide A aglycone in a 6.3% overall yield, and the spectroscopic data acquired for the synthetic product was consistent with the analogous data for the aglycone of **12a** synthesized by Willwacher and Fürstner.

**Table 1:** Comparative ^13^C NMR data (CDCl_3_, 175 ^†^ and 150 ^‡^ MHz) reported by McPhail and co-workers [18] for naturally-occurring **16**; by Willwacher *et al.* [20,21] for synthetic **12a**, **12b** and **16**; and by Xu, Ye and co-workers [19] for synthetic **12a** and **16**.

Carbon	Naturally-Occurring 16 [18] ^†^	Synthetic 12a [21] ^‡^	Synthetic 12b [21] ^‡^	Synthetic 12a [19] ^‡^	Synthetic 16 [19] ^‡^	Synthetic 16 [20] ^‡^
1	167.4	167.3	166.8	167.1	167.4	167.4
2	123.1	123.1	123.6	122.9	123.0	123.1
3	147.1	146.3	146.1	146.2	147.2	147.1
4	38.8	38.5	39.5	38.2	38.8	38.8
5	73.9	73.4	73.9	73.2	73.9	73.9
6	37.6	36.7	38.2	36.4	37.6	37.6
7	73.1	72.8	72.7	72.6	73.1	73.1
8	39.7	39.3	39.2	39.0	39.7	39.7
9	72.5	73.1	73.2	72.9	72.5	72.5
10	43.1	42.9	43.5	42.6	43.1	43.1
11	34.2	32.8	34.1	32.6	34.2	34.2
12	141.5	140.9	141.3	140.6	141.6	141.5
13	123.9	123.8	124.9	123.6	123.9	123.9
14	131.3	130.5	130.6	130.3	131.3	131.3
15	126.9	126.5	126.2	126.3	127.0	126.9
16	31.1	31.2	31.0	31.0	31.1	31.1
17	81.0	81.3	81.8	81.1	81.0	81.0
18	37.3	37.1	36.9	36.9	37.4	37.4
19	36.8	36.0	36.4	35.8	36.8	36.8
20	83.2	82.7	82.1	82.5	83.2	83.2
21	73.0	73.4	73.3	72.3	73.0	73.1
22	34.1	34.1	34.7	33.9	34.1	34.1
23	72.3	72.5	74.0	72.3	72.3	72.3
24	66.1	65.7	65.7	65.6	66.1	66.1
25	18.3	20.1	22.0	19.9	18.3	18.3
26	14.5	14.7	14.9	14.6	14.6	14.5
1′	94.2	94.0	94.1	93.7	94.2	94.2
2′	80.8	80.9	80.9	80.6	80.8	80.8
3′	71.7	71.7	71.6	71.4	71.7	71.7
4′	74.3	74.2	74.2	74.0	74.3	74.3
5′	68.1	68.2	68.2	67.9	68.1	68.1
6′	17.7	17.7	17.7	17.5	17.7	17.7
7′	59.1	59.2	59.1	59.0	59.2	59.1

**Table 2:** Comparative ^1^H NMR data (CDCl_3_, 700 ^†^ and 600 ^‡^ MHz) reported by McPhail and co-workers [18] for naturally-occurring **16** and by Willwacher *et al.* [20,21] for synthetic **12a** and **16**.

	Naturally-Occurring 16 [18] ^†^			Synthetic 12a [21] ^‡^			Synthetic 16 [20] ^‡^		
No.	δ (ppm)	mult.	*J* (Hz)	δ (ppm)	mult.	*J* (Hz)	δ (ppm)	mult.	*J* (Hz)
1									
2	6.01	dd	15.5, 1.2	5.92	dt	15.6, 1.5	6.01	dt	15.5, 0.8
3	6.97	ddd	15.2, 10.4, 4.6	7.02	ddd	15.5, 8.6, 5.5	6.96	ddd	15.3, 10.4, 4.9
4a	2.36	m		2.34	dddd	15.2, 6.5, 5.6, 1.8	2.36	m	
4b	2.39	ddd	14.1, 10.6, 10.6	2.46	dddd	15.2, 8.6, 3.7, 1.2	2.39	ddd	13.9, 10.8, 10.7
5	3.36	dddd	11.4, 11.4, 2.3, 2.3	3.42	m		3.37	m	
6a	1.20	m		1.26	m		1.20	m	
6b	2.02	dddd	12.6, 4.4, 2.3, 1.6	1.94	ddt	12.0, 4.6, 1.9	2.02	dddd	12.1, 5.6, 2.3, 1.6
7	3.82	dddd	11.1, 10.5, 4.4, 4.4	3.77	m		3.82	dddd	11.3, 10.6, 4.8, 4.5
8a	1.22	m		1.22	m		1.22	m	
8b	1.87	m		1.84	dddd	12.5, 4.2, 1.9, 1.9	1.87	dddt	13.2, 7.8, 5.3, 1.9
9	3.32	dddd	11.2, 11.2, 2.2, 2.2	3.33	m		3.31	tt	10.7, 2.1
10a	1.21	ddd	15.2, 9.6, 2.2	1.27	m		1.21	m	
10b	1.51	ddd	15.2, 11.2, 3.7	1.69	ddd	14.1, 9.1, 5.1	1.52	ddd	14.1, 11.1, 3.3
11	2.37	dqd	9.6, 6.5, 3.7	2.44	m		2.37	m	
12	5.45	dd	14.8, 9.7	5.61	dd	15.2, 7.6	5.44	dd	14.9, 9.9
13	6.28	dd	14.8, 9.7	6.22	ddt	15.2, 10.8, 1.0	6.27	dd	14.8, 11.1
14	6.05	dd	10.9, 10.9	6.01	tt	10.8, 1.8	6.05	dd	10.9, 10.9
15	5.28	ddd	10.8, 10.8, 5.6	5.27	ddd	10.8, 8.3, 7.5	5.28	dt	10.8, 5.6
16a	1.88	m		2.14	dddd	14.8, 6.8, 5.1, 1.9	1.88	m	
16b	2.28	ddd	13.1, 11.4, 11.4	2.29	dtd	14.8, 8.5, 1.6	2.25	m	
17	3.98	ddd	11.1, 8.1, 1.8	4.03	ddd	8.6, 7.2, 4.9	3.98	ddd	10.9, 8.5, 1.7
18	2.52	dddq	12.0, 7.0, 7.0	2.43	m		2.52	dddq	12.3, 7.0, 7.0, 6.9
19a	1.17	ddd	11.9, 11.9, 10.3	1.28	m		1.17	ddd	12.2, 12.1, 10.2
19b	2.01	ddd	12.2, 7.0, 5.6	2.04	dt	12.3, 6.7	2.01	ddd	11.8, 7.1, 6.0
20	3.63	m		3.71	ddd	8.4, 8.2, 6.7	3.63	m	
21	3.42	ddd	11.1, 8.8, 1.8	3.45	m		3.42	ddd	11.2, 8.9, 1.8
22a	1.46	ddd	14.1, 11.1, 1.9	1.54	ddd	14.4, 10.5, 2.5	1.46	ddd	14.2, 11.3, 1.9
22b	1.76	ddd	13.9, 11.7, 1.8	1.77	ddd	14.4, 10.8, 2.0	1.76	ddt	12.8, 12.6, 1.5
23	5.23	dddd	11.7, 4.9, 2.9, 1.9	5.24	m		5.23	dddd	11.6, 5.1, 3.1, 2.0
24a	3.61	m		3.65	m		3.61	m	
24b	3.81	dd	12.1, 2.9	3.78	dd	12.1, 3.3	3.79	m	
25	0.85	d	6.6	1.00	d	6.7	0.85	d	6.6
26	1.03	d	6.9	0.98	d	7.0	1.02	d	7.0
1′	5.02	d	1.1	5.02	d	1.5	5.02	d	1.1
2′	3.40	dd	3.8, 1.4	3.40	dd	3.8, 1.5	3.40	dd	3.4, 1.5
3′	3.68	m		3.69	m		3.68	td	9.8, 3.7
4′	3.34	dd	9.4, 9.4	3.34	t	9.4	3.34	dd	10.5, 9.3
5′	3.62	m		3.63	dd	9.4, 6.1	3.62	dd	9.9, 5.9
6′	1.27	d	6.3	1.28	d	6.3	1.26	d	6.3
7′	3.45	s		3.46	s		3.45	s	
OH-1′				2.45–2.33			2.69	br s	
OH-2′				2.56–2.33			2.31	br s	
OH-3′	2.24	s		2.44–2.34			2.35	m	
OH-4′	1.54	s		2.78–2.64	br s		2.53	br s	

**Figure 5:**
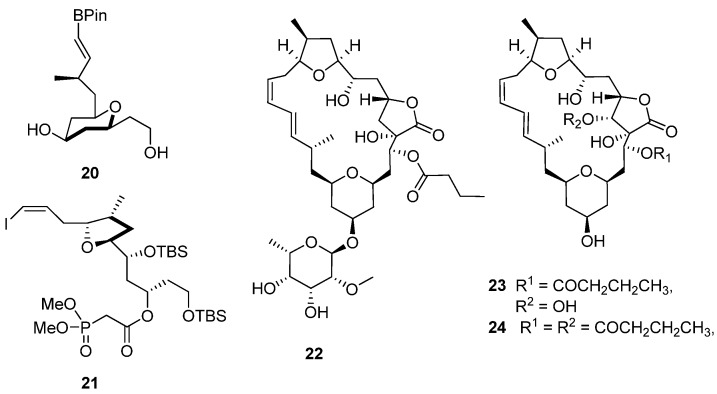
Chemical structures of compounds **20**–**25**.

A second synthesis of **12a** by Xu, Ye and co-workers [19] was published in Angewandte Chemie International Edition shortly after Willwacher and Fürstner’s synthetic communication appeared in the same volume of the journal. Approaching the synthesis of the 24-membered macrocycle via a different route to that used by Willwacher and Fürstner; Xu, Ye and co-workers initially constructed the two sub-units (**20** and **21**, Figure 5) with Prins cyclization, providing diastereoselective access to the tetrahydropyran moiety in **20** and a Rychnovsky–Bartlett cyclization generating the tetrahydrofuran ring in **21**. As anticipated from the retrosynthetic analysis that guided this synthetic approach, both subunits were successfully assembled into the aglycone via Suzuki coupling and Horner–Wadsworth–Emmons macrocyclization. The synthesis of **12a** was concluded with the addition of a protected rhamnose moiety to the mandelalide aglycone via a Kahne glycosylation reaction followed by a single-step collective removal of the silyl protecting groups. Xu, Ye and co-workers also identified the incompatibility of the NMR datasets acquired for their synthetic product and naturally-occurring mandelalide A, including the significant chemical shift differences associated with the C11 and C25 carbon atoms (Table 1 and Table 2). However, from direct comparison of the opposite configurations of the stereogenic centers in the northern hemisphere of **12a** and **17**, they correctly postulated that, assuming **12a** and **17** share a common biogenesis, the differences in absolute configuration would probably be confined to this part of the molecule and not C11, to which an *S* configuration was assigned in both **12a** and **17**. The convergent synthetic approach to **12a** by Xu, Ye and co-workers enabled them to synthesize the diastereomer, **16**, with opposite configurations at positions C17, C18 C20, C21 and C23 to those initially reported for **12a** [18] and consistent with the configurations assigned to the analogous chiral centers in **17** [19]. Willwacher *et al.* also recently reported a further synthesis of **16** [20] and confidently postulated revised structures for mandelalide B–D **(22**–**24**, Figure 5). Comparison of the NMR data of naturally-occurring mandelalide A with those of **16** (Table 1 and Table 2) confirmed that these two compounds were identical and that any uncertainties around the correct structure of mandelalide A had been successfully resolved.

Although the chemical structure of mandelalide A has now been unequivocally established as **16**, the inconsistency in the cytotoxicity data reported for this compound remains unresolved. McPhail and co-workers reported that the naturally-occurring mandelalides A and B possessed potent cytotoxicity against human NCI-H460 lung cancer cells (IC_50_ 12 and 44 nM, respectively) and Neuro-2A neuroblastoma cells (IC_50_ 29 and 84 nM, respectively) [18]. These results are at variance with the reported lack of cytotoxicity exhibited by synthetic **16** when screened against a panel of ten cancer cell lines of different histological origin by Xu, Ye and co-workers [19] (Table 3). Willwacher *et al.* also noted the negligible cytotoxicity of **16** against cancer cell lines with the exception of a single human breast carcinoma cell line [20] (Table 3).

**Table 3:** Comparative IC_50_ data (μM) reported by McPhail and co-workers [18] for naturally-occurring **16** and by Xu, Ye and co-workers [19] and Willwacher *et al.* [20] for synthetic **16**.

Cell Line	Histological Origin	Naturally-Occurring 16 [18]	Synthetic 16 [19]	Synthetic 16 [20]
Neuro-2A	Neuro	0.044		
NCI-H460	Lung	0.012		
H1299	Lung		25.5	
PC-3	Prostate		108.7	
PLC/PRF/5	Liver		140.6	
MHCC97L	Liver		>500	
HeLa	Cervix		249.0	
SH-SY5Y	Brain		>500	
HCT 116	Colon		>500	
HT-29	Colon		>500	>1000
MCF7	Breast		271.5	
MDA-MB-361-DYT2	Breast			0.041
N87	Stomach			0.206

## 4. Bisindole Alkaloid Inhibitors of Methicillin-Resistant *Staphylococcus aureus* Pyruvate Kinase from the South African Marine Sponge *Topsentia* sp.

Methicillin-resistant *Staphylococcus aureus* (MRSA), euphemistically also referred to as the “super bug”, was initially encountered in public healthcare facilities and remains a significant cause of mortality in these facilities. MRSA is no longer confined to healthcare facilities and has been increasingly reported from the general population and domestic livestock worldwide [25,26,27]. Annually, MRSA accounts for *ca.* 94,000 infections and 18,000 deaths in the USA and 150,000 infections in the EU [27]. There are no available MRSA mortality data from Southern Africa. However, a 2015 study conducted in three South African academic hospitals reported a MRSA prevalence rate (MRSA infections as a % of all recorded *S. aureus* infections) of 36%, which is comparable to Israel (33.5%) Ireland (38.1%) and the U.K. (35.5%) [28]. The escalating infection and mortality rates associated with the ongoing spread of drug-resistant pathogenic bacteria, e.g., MRSA, are further exacerbated by the dearth of new antibiotics entering the clinic [29].

**Figure 6:**
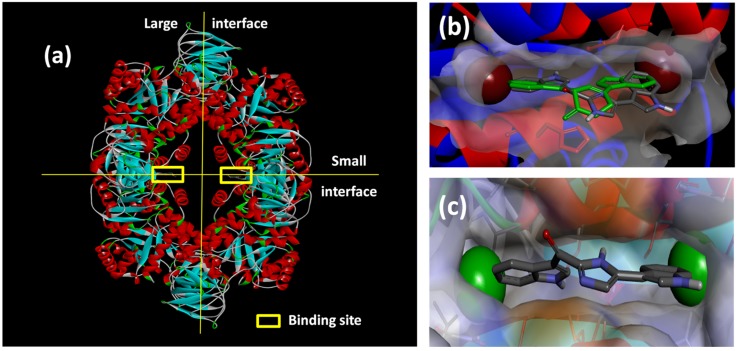
(**a**) X-ray structure of MRSA PK (PDB Accession Number 3T07) with the large and small interfaces and the *cis*-3,4-dihydrohamacanthin B binding sites indicated; (**b**) X-ray co-crystal generated diagram of **25** (green) in the *cis*-3,4-dihydrohamacanthin B binding site of MRSA PK with **26** (grey) overlaid in its highest scoring docked conformation with its bromines displayed as CPK models; (**c**) highest scoring docked conformation of the synthetic compound **33**, with its chlorines displayed as CPK models, in the MRSA PK *cis*-3,4-dihydrohamacanthin B binding site.

Paradoxically, the targeting of bacteria-specific proteins in new antibacterial drug development programs is problematic given the concomitant selective pressure that drugs, emerging from this classic drug discovery approach, exert on the pathogens, leading to the proliferation of drug-resistant bacterial strains [30]. Protein target-based antibiotic drug discovery is, however, not redundant. Contemporary genomic and proteomic studies of MRSA [31,32] have increased our understanding of the complex protein-protein interaction networks (interactomes) in this organism. The detailed mapping of interactomes has led to the identification of highly-connected hub proteins, which, given their centrality within the interactomes, are essential for mediating key cellular processes and sustaining MRSA viability [30,31]. Out of necessity, hub proteins are evolutionarily-conserved proteins, given the deleterious effect that mutations of hub proteins would have on the complex interactomes in which they play a key role [30]. Therefore, targeting hub proteins within the MRSA interactomes will minimize the potential for the emergence of drug resistance in MRSA and is a novel strategy for developing much needed new chemotherapeutic interventions against this drug-resistant pathogen [31]. Amongst the suite of hub proteins in a 608-protein interactome network (comprising 23% of the proteome in a hospital-acquired strain of MRSA), Zoraghi *et al.* identified pyruvate kinase (PK) as a suitable target for possible antibiotic drug discovery [30]. Catalyzing the rate-limiting irreversible conversion of phosphoenolpyruvate into pyruvate during glycolysis, pyruvate kinases are, not surprisingly, ubiquitous in both prokaryotes and eukaryotes. Fortuitously, the MRSA PK homotetramer (Figure 6a) has several possible lipophilic binding pockets that are absent in human PK orthologs, allowing potential selective inhibition of this enzyme target [33]. Initially, two parallel strategies were used to generate lead compounds to exploit the inhibition of this key enzyme. The first strategy involved the random screening of >900 marine invertebrate extracts, including those from South African marine invertebrates, for selective MRSA PK inhibition. The second rational drug design strategy coupled knowledge of the detailed structure of the MRSA PK enzyme binding site with contemporary computer-aided drug design techniques to generate new synthetic MRSA PK inhibitors. Both strategies are reviewed in more detail below.

**Figure 7:**
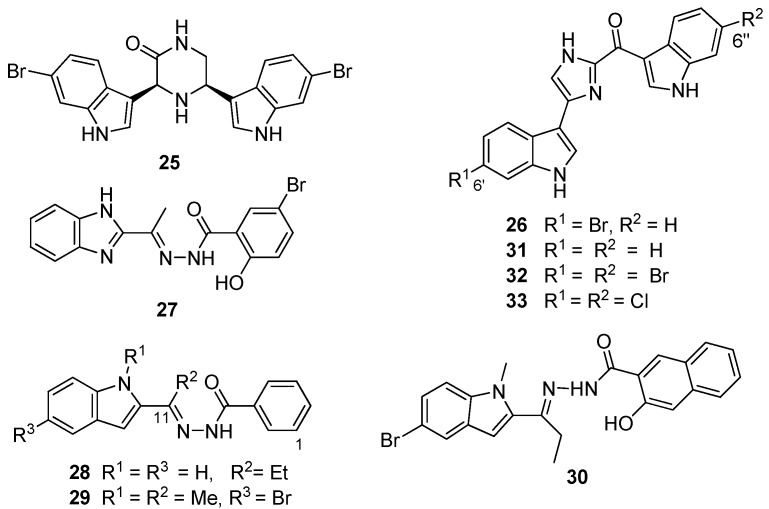
Chemical structures of compounds **25**–**33**.

The random screening of 968 marine invertebrate extracts, collected from seven different benthic marine environments around the world, [34], afforded only one extract that was active in the MRSA PK inhibition assay. The methanolic extract of the South African sponge, *Topsentia pachastrelloides* (Figure 1c), showed significant activity in the MRSA PK inhibition assay, and subsequent bioassay-guided fractionation of this extract yielded a cohort of four bisindole alkaloids of which, the two known metabolites *cis*-3,4-dihydrohamacanthin B (**25**, Figure 7) and bromodeoxytopsentin (**26**) proved to be the most active compounds (IC_50_ 16 and 60 nM, respectively). These two compounds also exhibited between 166- and 600-fold selectivity for MRSA PK when compared to similar inhibition data acquired from screening **25** and **26** against four human PK orthologs. X-ray crystallographic analysis of the co-crystallized *cis*-3,4-dihydrohamacanthin B-MRSA PK complex revealed that **25** was neither bound to the recognized activation nor allosteric effector binding sites on this enzyme, but was instead unexpectedly bound to two identical lipophilic binding sites on the small interface of the MRSA PK homotetramer [34].

The small interface in MRSA PK is postulated to be crucial for establishing the rigidity of MRSA PK necessary for catalytic activity, and the binding of either **25** or **26** to this region of the protein is therefore thought to induce flexibility and, subsequently, to reduce enzyme activity [33]. The symmetrical *cis*-3,4-dihydrohamacanthin B binding sites are characterized by two lipophilic pockets with an appropriate spatial arrangement to readily accommodate the bromine substituents of **25** and **26** (Figure 6b). In addition, the histidine residues (HIS365) on the neighboring parallel MRSA PK α-helices rearrange to anchor the indole rings through π-interactions [34,35]. Interestingly, sequence alignment between MRSA and human PK isoforms indicated that access to the analogous binding sites in human PK orthologs is hindered by a group of amino acids that effectively shield these sites from potential ligands [33]. This structural difference around the entrance to the binding sites is consequently thought to account for the greater selectivity of **25** and **26**, and related synthetic inhibitors *vide infra*, for MRSA PK over human PK orthologs [33,35].

From the preliminary screening of an *in silico* library, coupled with functional enzyme assays, Zoraghi *et al.* identified the benzimidazole compound IS-130 (**27**, Figure 7) as a potent MRSA PK inhibitor (IC_50_ 91 nM) with good specificity (>1000-fold) for MRSA PK over human isoforms, but with poor *in vivo* antibacterial activity at the cellular level against a methicillin-susceptible strain of *S. aureus* (MSSA) (minimum inhibitory concentration (MIC) >187 μg/mL) [30]. Nevertheless, the structural motif of **27** provided the starting point for a medicinal chemistry program aimed at improving selectivity, potency and antibacterial activity of potential MRSA PK inhibitors. Compound AM-168 (**28**) exhibited only slightly reduced potency (IC_50_ 126 nM) and substantially increased antibacterial activity (MIC 9.7 μg/mL), which was attributed by Zoraghi *et al.* to increased cell membrane penetration due to the increased lipophilicity imparted by the C11 ethyl substituent to this compound [30]. Accordingly, additional alkyl substitution, e.g., NSK5-15 (**29**), further enhanced antibacterial activity against several strains of MSSA and MRSA (MIC 1.4–5.8 μg/mL) with a further decrease in MRSA PK inhibitory potency (185 nM). Kumar *et al.* [33] extended Zoraghi *et al.*’s preliminary study and prepared a series of >70 compounds in which systematic structural changes were made to the heteroaromatic ring, the phenolic moiety and the central linker unit of the hit compound **27**. This series of compounds was screened against MRSA PK and methicillin-susceptible *S. aureus*, with Kumar *et al.* reporting varying levels of potency (IC_50_ 15–380 nM) and antibacterial activity (MIC 1–>194 μg/mL), respectively. Interestingly, co-crystallization of **27** and **28** with MRSA PK followed by X-ray analysis revealed that these compounds were also bound to the *cis*-3,4-dihydrohamacanthin B binding sites of the MRSA PK enzyme (Figure 6a) [33]. Unfortunately, Kumar *et al.*’s synthetic program did not shed any light on the structure activity relationships that might conclusively link potency (IC_50_) with antibacterial activity (MIC). Ultimately, *N*-methylindole (**30**) provided the best combination of *in vitro* MRSA PK inhibition (IC_50_ 79 nM) and antibacterial activity (MIC 1 μg/mL), possibly warranting further exploitation of **30** and analogous compounds as potential antibiotics effective against MRSA [33].

Veale *et al.* [35] used the chemical structure of the naturally-occurring hit compound **26** identified in the South African sponge extract as a starting point for an extensive ligand-receptor docking study of various analogs of **26** with the *cis*-3,4-dihydrohamacanthin B binding site. Postulating that a dihalogenated analog of **26** would better exploit the opportunities offered by the symmetrical *cis*-3,4-dihydrohamacanthin B binding site, in particular the two terminal lipophilic binding pockets, Veale *et al.* prepared the 6′, 6″dihalogenated (F, Cl, Br and I, Figure 7) analogues of **26** and the debrominated compound, deoxytopsentin (**31**), for a comparative MRSA PK inhibition study. The target halogenated synthetic compounds were readily accessed in reasonable overall yield (10%–32% over five steps) via the dehydrative cyclocondensation of the respective *N*-Boc-protected 6-halo-indolyl-3-glyoxals with ammonium acetate in ethanol, followed by thermolytic cleavage of the *N*-Boc groups. As expected, the MRSA inhibition activity of the non-halogenated compound **31** (IC_50_ 240 nM) was less active than the naturally-occurring monobrominated compound **26** (IC_50_ 60 nM), while both the dibrominated and dichlorinated (Figure 6c) analogs (**32** and **33**) were an order of magnitude more potent (IC_50_ 2 and 1.5 nM, respectively) than **26** coupled to improved selectivity for MRSA PK over the four human orthologs assayed [35].

**Figure 8:**
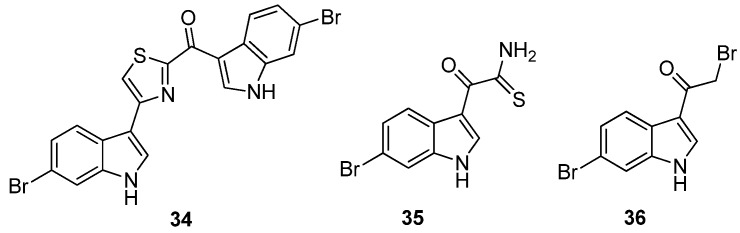
Chemical structures of compounds **34**–**36**.

Veale *et al.* further evaluated the importance of the imidazole ring to MRSA PK inhibition in dihalogenated bisindole alkaloids by preparing a similar series of dihalogenated bisindoles in which the imidazole ring was replaced by a thiazole moiety, e.g., **34** [36] (Figure 8). Coupling of α-oxo-1*H*-indole-3-thioacetamide (**35**) with the α-bromoketone (**36**) in a regiospecific Hantzsch thiazole ring formation reaction afforded the targeted halogenated bisindole thiazoles. The μM activity of the synthetic bisindole thiazoles (e.g., IC_50_ 5 μM for **35**) indicated that bioisosteric replacement of the imidazole ring with a thiazole had a negative impact on MRSA PK potency [36]. The antibacterial activity of both the synthetic bisindole imidazoles and bisindole thiazoles was not recorded.

**Figure 9:**
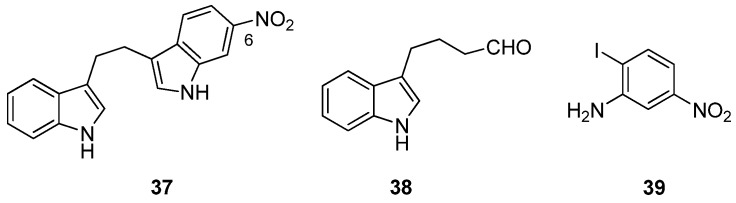
Chemical structures of compounds **37**–**39**.

Furthermore, acknowledging the significance of a bisindole motif to increased inhibition of MRSA PK, Sperry and co-workers have recently described the selective MRSA PK inhibition of a cohort of eleven, variously-substituted, synthetic 1,2-bis(3-indolyly)ethane compounds, e.g., **37** [37] (Figure 9). Compound **37**, accessed via palladium catalyzed heteroannulation of the aldehyde (**38**) and 1-iodo-2-amino-4-nitrobenzene (**39**) [38], was the most potent of the series (IC_50_ 0.9 μM) and exhibited a 20–106-fold selectivity for MRSA PK over four human PK isoforms. Replacing the C6 nitro substituent with chloro, nitrile, methoxy and methyl functionalities had a deleterious effect on MRSA PK inhibition, with the nitrile and methoxy analogs inactive and the chloro and methyl analogs two orders of magnitude less active (IC_50_ 272 and 294 μM, respectively) [37].

**Figure 10:**
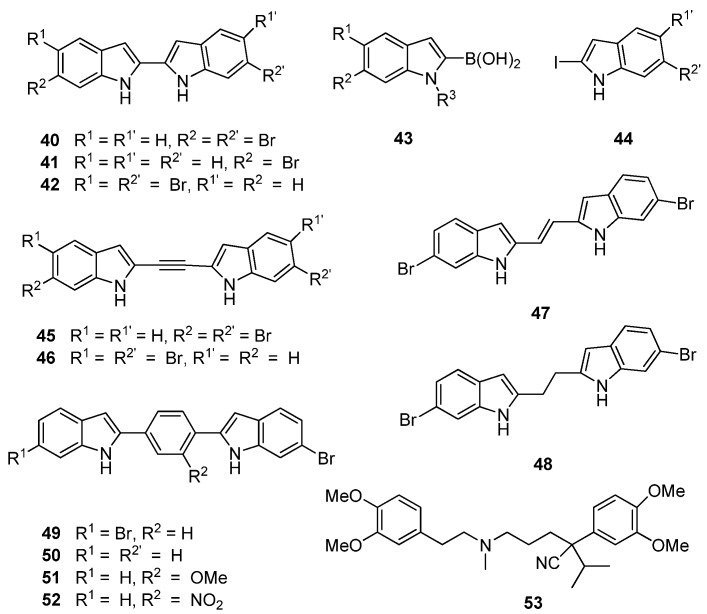
Chemical structures of compounds **40**–**53**.

Similarly, Kumar *et al.* [39] changed direction from their earlier medicinal chemistry program based on the putative MRSA PK inhibitor, **27**, to focus on potential bisindole inhibitors of MRSA PK in line with the chemical structures of the potent naturally-occurring bisindole MRSA PK inhibitors, **25** and **26**. Central to their strategy was varying the linker units between the indole rings in order to uncover the relationship between activity and indole orientation relative to the linker unit in addition to providing further insight into size constraints within the MRSA PK binding site. Their initial cohort of directly-linked 2,2′-biindoles (**40**–**42**, Figure 10) was synthesized through a Suzuki–Miyaura coupling of boronic acid precursor **43** and iodinated indole **44**. This cohort of compounds were generally found to potently inhibit MRSA PK at concentrations as low as 1 nM. Initial inhibitory data obtained by Kumar *et al.* supported previous observations made between deoxytopsentin analogues **26** and **32** that 6-6′ dibrominated bisindoles, such as 40 (IC_50_ 7 nM), display superior MRSA PK inhibition than a corresponding monobrominated analogue, e.g., **41** (IC_50_ 21 nM). However, the opposite trend was observed with regard to MIC values (16 and 2 μg/mL, respectively) against MSSA strains [39]. Interestingly, the 6,5′ dibrominated analogue (**42**) also displayed potent MRSA PK inhibition (IC_50_ 2.2 nM) coupled to a significantly improved MIC against *S. aureus* (0.3 μg/mL). The structure activity relationship of the linker group between indoles was further explored through insertion of acetylene, ethylene and ethyl moieties between the two substituted indole rings (**45**–**48**, Figure 10) using standard synthetic protocols. Similar MPSA PK inhibitory activity was observed between this group and the 2,2′-biindoles. However, MIC activity against *S. aureus* was generally lost, with the single exception of the 6,5′ dibrominated analogue **46**. An additional set of aryl linked bisindole analogues (**49**–**52**) were prepared with a view toward exploiting possible interactions with the aromatic histidine residues present in the binding site. The dibrominated compound (**49**) was found to be comparatively less active than compound **40**, while activity was restored with the mono-brominated compound (**50**), leading the authors to suggest that Compound **50** defines the maximum permissible length within the MRSA PK binding site. Interestingly, analogues of **50** featuring substitutions on the aryl ring (**51**, **52**) were found to be the most active in the series against MRSA PK (IC_50_ *ca.* 2 nM) while the nitro-containing compound **52** showed encouraging activity against *S. aureus* (MIC 2.0 μg/mL). While no conclusive rationale for the differences between MRSA PK inhibitory activity and MIC values was postulated, Kumar *et al.* determined, through co-administration of their synthetic compounds with the calcium channel blocker verapamil (**53**), that several bisindoles were actively removed from the cells via cellular efflux mechanisms, possibly accounting for the contrasting antibacterial activities observed in their study [39].

## 5. Conclusions

Interest from Pettit and others in the anti-cancer potential of **1** has remained undiminished for nearly three decades and is likely to continue for the foreseeable future. Realization of the true potential and possible further drug development of the cephalostatins has been hampered by access to commercially-viable synthesis of sufficient quantities of either **1** or similarly-bioactive congeners. Although accessible by laboratory synthesis, potential future drug development interest in **16** will only resume if conflicting cancer cell cytotoxicity data reported for naturally-occurring and synthetic **16** can be explained. The negative impact of drug-resistant pathogens, e.g., MRSA on human health is steadily increasing, and the need for new antibiotics against these pathogens is continually emphasized. Although the *cis*-3,4-dihydrohamacanthin B binding site of MRSA PK has been identified as a potential selective anti-biotic drug target, resolving the conundrum between potent MRSA PK inhibition and poor *in vivo* MRSA antibacterial activity will define the future of this approach to MRSA antibiotic drug discovery.
